# *Staphylococcus aureus* Clumping Factor A Remains a Viable Vaccine Target for Prevention of *S. aureus* Infection

**DOI:** 10.1128/mBio.00225-16

**Published:** 2016-03-08

**Authors:** Annaliesa S. Anderson, Ingrid L. Scully, Ed T. Buurman, Joseph Eiden, Kathrin U. Jansen

**Affiliations:** Pfizer Vaccine Research and Development, Pearl River, New York, USA

## Abstract

In a recent article, X. Li et al. [mBio 7(1):e02232-15, 2016, http://dx.doi.org/10.1128/mBio.02232-15] investigate the utility of a vaccine composed of the *Staphylococcus aureus* protein clumping factor A (ClfA) in protecting mice from *S. aureus* infection. ClfA, one of the first proteins to be identified as a potential vaccine antigen for *S. aureus* prophylaxis, is currently a component of several investigational vaccines. The authors conclude that ClfA may not be effective for *S. aureus* prophylaxis. In contrast, previously published papers reporting positive data suggested that ClfA was potentially an important vaccine target to prevent invasive *S. aureus* disease. This commentary addresses the observed differences between the findings of Li et al. and those from other publications, highlighting the importance for preclinical vaccine antigen assessments to reflect the biological role of said antigen in virulence and, consequently, the importance of choosing appropriate preclinical disease models to test such antigens.

## COMMENTARY

*Staphylococcus aureus* is a clinically relevant Gram-positive organism that is carried asymptomatically in the nares of 20 to 50% of the general population (1). However, upon a breach of skin or mucosal barriers, it can cause a wide spectrum of disease, ranging from relatively mild skin infections, such as carbuncles, to life-threatening wound and bloodstream infections (2). *S. aureus* is recognized as a leading cause of morbidity and mortality in both health care-associated and community settings. *S. aureus* infections following surgery carry particularly high mortality rates, and survivors require, on average, an additional 13 to 17 days of hospitalization, significantly increasing health care costs (3). The burden of *S. aureus* disease is exacerbated by the prevalence of antibiotic-resistant *S. aureus*, such as methicillin-resistant *S. aureus* (MRSA) (4). Infection control measures have been effective in stabilizing rates of MRSA (5, 6); however, the overall burden of disease remains high, emphasizing the need for effective prophylactic vaccines for populations at risk of disease.

No licensed vaccine is currently available for the prevention of *S. aureus* disease, although some vaccines are in clinical trials (7, 8). The *S. aureus* vaccine field has been hampered by two notable vaccine failures, StaphVax, comprised of capsular polysaccharide conjugates, and V710, comprised of iron surface determinant B (IsdB). It is unclear whether the respective antigens selected, the single-antigen approach, or other factors such as patient population or manufacturing issues led to the lack of positive outcome (9). While many vaccines that protect against bacterial diseases are based on single antigens (e.g., capsule for *Haemophilus influenzae* type B and *Streptococcus pneumoniae*), there is already preclinical evidence that a multiantigen approach may likely be required to confer protection against *S. aureus* (10). Furthermore, *S. aureus* has a wide variety of virulence mechanisms ranging from toxin elaboration to adhesion to host factors to nutrient scavenging, and it causes a range of diseases where more than one of these virulence mechanisms plays a role. Therefore, it is possible that the targeting of a single virulence factor may never be fully efficacious either preclinically or clinically for *S. aureus*.

Li et al. present a large body of *in vivo* and *in vitro* data interrogating clumping factor A (ClfA) as a possible vaccine antigen (11). ClfA was one of the first *S. aureus* virulence factors validated preclinically (12–17), and as a direct result of these preclinical findings, ClfA antigens were licensed by several companies for inclusion in current multiantigen vaccine approaches in clinical development (7, 18). Importantly, the Schneewind laboratory and others have found that ClfA, which functions as an adhesion factor, appears to exhibit its primary effect early during infection (19). In animal models where *S. aureus* is delivered systemically, such as in sepsis and in peritoneal infections, the initial adhesion events that require ClfA function are bypassed, corroborating the findings of Li et al. that immunization with ClfA shows little effect in these models.

A rationally designed approach for *S. aureus* vaccine development needs to target multiple virulence pathways. To evaluate these virulence mechanisms, there must be a method of verifying a relevant host immune response, either in animal models or in *in vitro* assays and ideally in both. Animal models of infection can contribute a great deal to the understanding of pathogenic mechanisms, as long as their limitations are clearly understood. Once an antigen has been validated in a preclinical model, clinical immunology assays must be developed to examine the performance of the vaccine in humans and to potentially define a correlate of protection. It is important to demonstrate that the measurement of the immune responses reflects functional responses, i.e., responses that can inhibit the mechanism of action of the virulence factor or vaccine target.

In the case of Gram-positive pathogens, opsonophagocytic activity (OPA) assays, which measure the ability of antibodies to induce the uptake and killing of encapsulated bacteria, are established standards to measure functional antibody responses to bacterial capsules that are important virulence factors. In the case of *S. aureus*, anticapsular antibodies have been shown to elicit bacterial killing (20, 21). However, for subcapsular antigens, there is little evidence that these antigens induce a true bacterial killing response. Instead, understanding their mechanism of action *in vivo* allows the development of appropriate assays to ascertain that the elicited immune response “neutralizes” the action of the virulence factor (22). In the case of ClfA, opsonophagocytosis-mediated killing does not address its virulence mechanism, and true OPA has rarely been described. Domanski et al. demonstrated that antibodies directed against ClfA could induce the uptake of ClfA-coated beads by human polymorphonuclear leukocytes (PMNs), but killing of the pathogen was not assessed (23). Li et al. described detection of OPA using purified rabbit antibodies generated with a proprietary adjuvant provided by GlycoVaxyn and reported a low level of opsonophagocytic killing activity at the highest concentration of antibodies. Likewise, very little bacterial killing was observed with ClfA immune mouse sera. Similarly low levels were observed using a high concentration of ClfA-specific affinity-purified human serum (24). This is in contrast to what is reported with capsular polysaccharide conjugate-induced responses, where high levels of killing are seen (18).

The primary virulence function of ClfA is to adhere to human fibrinogen during the initiation of infection (15, 25, 26), and an appropriate assay to measure the inhibition of that binding is required for relevant antibody assessment. A fibrinogen binding inhibition (FBI) assay was developed to monitor such functional antibody responses (27). This assay measures the ability of serum from vaccinated individuals to prevent binding of *S. aureus* to human fibrinogen. Clinical *S. aureus* strains are ideally used in these assays to ensure the presence of a full complement of adhesins, capsular polysaccharide, and protein A. Hawkins et al. examined the immune responses of both rhesus macaques and human volunteers upon immunization with ClfA (27). Human subjects all had preexisting binding antibodies to ClfA; however, these antibodies were unable to prevent the binding of *S. aureus* to fibrinogen. Once the human subjects were immunized with a vaccine that included ClfA, however, a robust functional immune response that prevented clinical *S. aureus* isolates from binding to fibrinogen was observed. This initially surprising finding was further explored in rhesus macaques, revealing that immune exposure to *S. aureus* resulted in the generation of nonfunctional antibodies, but when the immune response was elicited by a ClfA-containing vaccine, functional antibodies were generated (27). To establish a correlation between ClfA FBI titers and the *in vivo* neutralization of ClfA activity, a system of ectopic expression of ClfA in *Lactococcus lactis* was developed. Though this was an artificial system, it provided a clean background for the specific assessment of functional ClfA responses (28). This afforded valuable insight into what level of antibody response may reflect a correlate of protection and validated the outcome of the *in vitro* serological FBI assay as clinically relevant. In this way, sera and/or antibodies with the minimum detectable FBI activity were shown to neutralize ClfA activity *in vivo* of suspensions of 10e9 ClfA-expressing bacteria, thus identifying a preclinical serological correlate for ClfA (28). Li et al. were able to demonstrate the induction of FBI titers; however, they used a genetically modified strain that did not express the IgG antibody adhesion factor protein A, so it is difficult to assess the results in the context of the observations of Scully et al. (28).

In their analysis of ClfA as a potential vaccine antigen, Li et al. (11) may not have fully considered the molecular basis for the virulence activity of ClfA and the assessment of immune responses to ClfA, thereby contributing to their conclusion that ClfA would be a poor vaccine candidate for humans. The mechanism by which ClfA contributes to virulence (binding to human fibrinogen) has been clearly and carefully elucidated (16, 19, 25, 27, 28). Therefore, the prevention of the use of ClfA by invading *S. aureus* to bind fibrinogen (which is ubiquitous in the host) is an important means of interrupting pathogenicity, as demonstrated by the body of preclinical data generated by multiple laboratories and in diverse animal models. In summary, multiple antigens targeting independent virulence pathways are likely required for a vaccine to be effective against *S. aureus* disease. In addition, an animal model(s) that reflects the virulence mechanism of the antigen of interest is required for vaccine target evaluation. Finally, it is critical to measure whether a vaccine candidate elicits the desired immune responses to inhibit a given virulence factor. These points would indicate that the conclusions drawn by Li et al. may be overly simplistic and ultimately do not argue against the potential usefulness of ClfA as an antigen for inclusion in an investigational multiantigen vaccine designed to prevent human *S. aureus* disease.
